# Optic Nerve Hemangioblastoma: A Case Report

**DOI:** 10.1155/2012/915408

**Published:** 2012-04-24

**Authors:** Holly Zywicke, Cheryl Ann Palmer, Michael S. Vaphiades, Kristen O. Riley

**Affiliations:** ^1^Department of Surgery, Division of Neurosurgery, University of Alabama at Birmingham, Birmingham, AL 35294, USA; ^2^Department of Pathology, Division of Neuropathology, University of Alabama at Birmingham, 1960 6th Avenue South, PD6A 175E, Birmingham, AL 35294, USA; ^3^Department of Ophthalmology, University of Alabama at Birmingham, Birmingham, AL 35294, USA

## Abstract

Hemangioblastomas are World Health Organization (WHO) grade I tumors of uncertain histologic origin. These central nervous system tumors are most often found in the posterior fossa, brainstem, and spinal cord. There are fewer than 20 reported cases of optic nerve hemangioblastomas in the literature. We present a patient with visual decline found to have a mass arising from within the posterior orbital canal that grossly involved the optic nerve sheath. Neuropathologic evaluation showed hemangioblastoma. Although not a common tumor in this location, consideration of hemangioblastoma in the differential diagnosis is important as they can have a more aggressive course than other tumors of this region and have a detrimental effect on visual prognosis.

## 1. Introduction

 Hemangioblastomas are exclusive to the central nervous system (CNS) accounting for two percent of all primary intracranial tumors and approximately ten percent of adult posterior fossa tumors [1]. They are considered benign, slow-growing tumors that often contain both solid and cystic components [2, 3]. They occur either sporadically or in association with von Hippel-Lindau (VHL) disease. VHL-disease is a heritable systemic syndrome that manifests in the CNS with multiple intracranial and retinal hemangioblastomas. Although surgical outcomes are similar for both sporadically occurring and VHL disease-associated hemangioblastomas, patients with VHL disease have a higher morbidity and mortality secondary to multifocal disease [4, 5]. Therefore, assessment for the presence of VHL disease is important when a CNS hemangioblastoma is identified.

Tumors that involve the optic nerve are considered primary, arising from the optic nerve or its coverings, or secondary, deriving from structures outside of the optic nerve [6]. Although rare, optic nerve hemangioblastomas have been reported in the literature [7–10]. We discuss the importance of the consideration of hemangioblastoma in the differential diagnosis for tumors of the optic nerve to allow for appropriate operative planning, management, and prognosis.

## 2. Case Report

### 2.1. History

A 50-year-old right-handed woman noted progressive visual loss in the left eye for two years. Further questioning revealed complaints of fatigue, dry skin, brittle nails, and thinning hair.

### 2.2. Examination

Her visual acuity was 20/50 in the right eye and 20/70 in the left eye. Visual fields were full to confrontation. Her pupillary examination, ocular motility and fundus examinations were normal. The remainder of her neurological and general physical examinations were unremarkable.

### 2.3. Imaging

**Figure 1:**
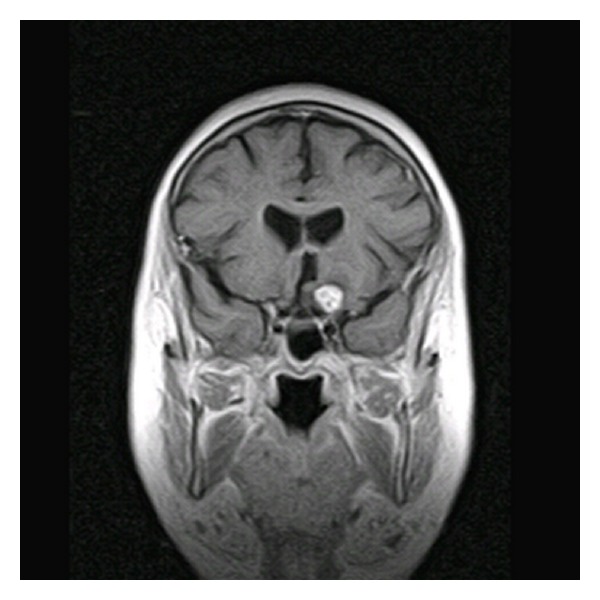
Axial MRI demonstrates avidly enhancing mass in the left paraclinoid region.

Magnetic resonance imaging (MRI) showed a left-sided 1 centimeter by 1 centimeter enhancing lesion arising from the posterior orbital canal with extension into the paraclinoid region (Figure 1). The mass appeared to compress the left optic nerve. The enhancement pattern, location, and shape of the mass suggested a meningioma.

### 2.4. Surgery

A left frontotemporal craniotomy was performed. Tumor was visualized along the skull base with attachments to the orbital roof and clinoid. Tumor encased the optic nerve demonstrating a somewhat soft consistency with exceptional vascularity. Partial resection was obtained with tumor left in the optic canal anteriorly where it surrounded the optic nerve. It appeared to arise from the optic nerve sheath grossly.

### 2.5. Neuropathology

**Figure 2:**
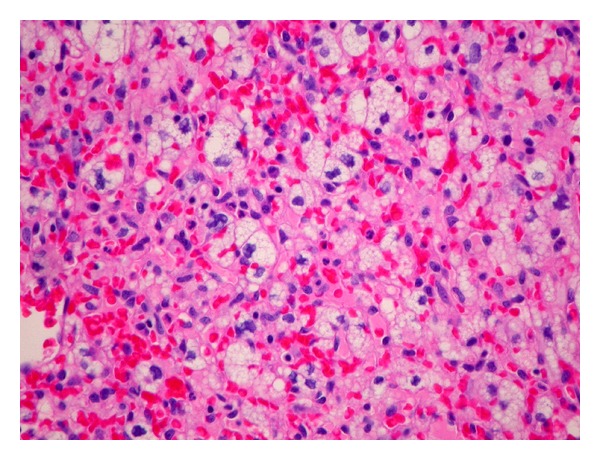
H&E photomicrograph reveals a markedly vascular tumor with lipidized stromal cells (×200).

**Figure 3:**
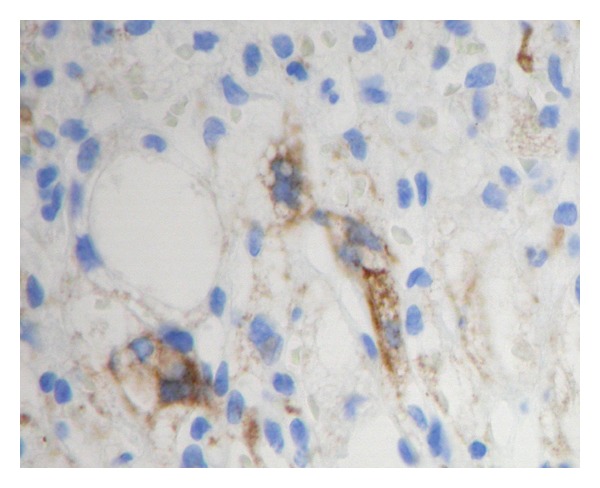
Inhibin immunohistochemistry was positive in scattered tumor cells throughout the neoplasm (×400).

The tumor displayed abundant vascularity with both thick-walled and thin-walled large vessels and an extensive capillary network. Stromal (tumor) cells were arranged in lobular patterns and displayed abundant foamy lipid with irregular, hyperchromatic nuclei (Figure 2). Mitoses were not seen, and necrosis was absent. Scattered reactive inflammatory cells were present. Inhibin immunohistochemistry (Dako, Carpenteria, CA, USA) was focally positive in the stromal (tumor) cells but negative in the endothelial cells (Figure 3). CD10, RCC, and epithelial membrane antigen immunostains were negative in the specimen.

### 2.6. Postoperative Course

Postresection MRI revealed a small region of nonenhancing residual tumor within the optic canal. Visual acuity had now worsened in the left eye to hand movement with associated visual field loss and a relative afferent pupillary defect. The vision in the right eye was unchanged. Adjuvant radiation therapy was not pursued. Complete neuraxis and abdominal cavity imaging was without evidence of systemic VHL disease.

## 3. Discussion

Optic nerve tumors are considered primary or secondary [11]. Secondary tumors are malignancies that spread from the hematopoietic system and distant organs to invade the optic nerve [12]. Primary optic nerve tumors arise from either the optic nerve proper or its coverings [11]. Those that arise from the optic nerve proper are classified as intrinsic and include benign or malignant optic gliomas [11]. Optic nerve sheath meningiomas, gangliogliomas, medulloepitheliomas, and hemangioblastomas are the more common extrinsic optic nerve tumors [6, 11].

Intrinsic and extrinsic optic nerve tumors commonly result in progressive vision loss [6, 11]. While intrinsic tumors generally cause visual loss through destruction of the optic nerve fibers, extrinsic tumors produce visual decline either through direct compression of the optic nerve or disruption of the optic nerve vasculature [11, 12]. Extrinsic tumors that cause visual decline via compression of the optic nerve are often considered for surgery. During resection care must be taken to avoid both damage to and devascularization of the optic nerve as its unique anatomy and blood supply place it at high risk for injury.

As the optic nerve exits the globe posteriorly en route to the optic chiasm, it travels through both the orbit and optic canal. Unlike the rest of the optic nerve, the intraorbital and intracanalicular portions are poorly vascularized [13]. Only two vessels, the ophthalmic artery and superior hypophyseal arteries, provide blood to this region [13]. As a result, both areas are susceptible to ischemia. The intracanalicular portion, however, is especially vulnerable due to the narrow diameter of the optic canal [13, 14]. The superior hypophyseal artery provides two to three trunks to the intracanalicular optic nerve that then branch extensively to form a dense internal capillary network [14]. These vessels are of very small caliber, which contributes to their increased susceptibility to ischemic injury. Additionally, the lack of contributing vessels allows for minimal opportunity for the development of collaterals. Hence, as the optic nerve relies primarily on its microscopic internal vascular network for blood supply, the area is sensitive to vascular injury associated with compression or increased intracranial pressure. Therefore, when tumors are located in this area, there is higher risk for visual loss.

In this case, not only did tumor location put the patient at increased risk for visual loss, but also the type of tumor provided additional threat to visual morbidity. Hemangioblastomas are highly vascular CNS tumors. To develop their highly vascular internal network, hemangioblastomas recruit blood vessels from surrounding structures [15]. Hemangioblastomas have also been shown to induce hypertrophy in their feeding vessels [16], which can cause a steal syndrome phenomenon. In this case, proximity to the optic nerve in conjunction with the relatively scant blood supply to the intracanalicular optic nerve may have, in part, accounted for the visual loss observed. This hypervascularity can also cause excessive bleeding during resection that leads to obscuration of the operative field and promotes damage to the surrounding tissue [17]. Preoperative embolization has been shown to minimize blood loss leading to a reduction in complications attributed to excessive blood loss [17–19]. Embolization was not an option in this case as the tumor and optic nerve had a shared blood supply and would likely have resulted in complete visual loss. Therefore, when considering tumor types in the differential diagnosis of optic nerve lesions, it is important to include hemangioblastoma as there is higher risk of visual morbidity associated with their inherent vascular nature.

Besides vascularity and location, hemangioblastomas may have increased morbidity secondary to their association with VHL disease. The majority of this morbidity comes from development of tumors and cysts of the visceral organs including renal cell carcinoma (RCC), pheochromocytoma, and pancreatic cysts [3]. Although hemangioblastoma is the most common CNS lesion in VHL disease, these other tumors can metastasize to the intracranial compartment [5]. Pathologically, the clear cytoplasm of RCC can resemble the lipid-laden stroma of hemangioblastoma and may require further evaluation. Fat stains, such as an Oil Red O can confirm lipid content if performed prior to tissue fixation, which dissolves the lipid substances. Immunohistochemical studies may also be necessary. These can include inhibin, a peptide hormone belonging to the transforming growth factor-beta family, which is useful in distinguishing hemangioblastoma from metastatic RCC [20]. Hemangioblastomas, as was the case in our specimen, are positive for inhibin; RCCs are generally negative. Hence, with the diagnosis of CNS hemangioblastoma, VHL disease should be considered to evaluate for systemic disease and rule out other VHL disease-related CNS lesions.

Although optic nerve hemangioblastomas pose significant risk to vision, the overall morbidity and mortality of nonoptic nerve CNS hemangioblastomas are markedly low [4, 5, 19]. In fact, tumor location has not been found to predict a worse prognosis, rather multiplicity at time of diagnosis and development of new CNS lesions over time correlate with higher morbidity and mortality [1, 4]. This is important as nearly 40% of all hemangioblastomas occur in association with VHL disease and those that do have higher rates of multifocality and new lesion formation [1, 5]. VHL hemangioblastomas also carry higher morbidity and mortality rates due to multisystem involvement. Systemic manifestations of VHL disease include visceral lesions such as RCC, pheochromocytoma, and pancreatic cysts. 

Cardiovascular disease with subsequent stroke and heart attack may also occur as a result of angiomatosis. Therefore, when a CNS hemangioblastoma is identified, it is important to evaluate the patient for the presence of VHL disease. The National Institutes of Health (NIH) screening protocol includes enhanced MRI of entire neuraxis, urinary catecholamine evaluation, ophthalmoscopy, computed tomography, and ultrasound of the abdomen [21, 22]. Patients diagnosed with VHL disease require periodic follow-up to evaluate for new manifestations of the disease [21].

The treatment of choice for CNS hemangioblastomas is surgery with the goal of complete resection. Gross total resection is often curative; however, vascularity or critical location can limit the extent of resection possible. Even with complete tumor removal, recurrence is noted in up to 25% of cases [23]. Subtotal resection is associated with higher rate or recurrence [23]. As a result, radiation therapy has been gaining favor in the treatment of hemangioblastoma. The role of conventional radiation is more limited in use, usually being applied for multicentric CNS disease [24, 25]. Stereotactic radiosurgery (SRS), however, is becoming increasingly popular as either primary treatment in cases of multifocal CNS hemangioblastomas or for lesions whose locations are not amenable to surgical resection [26, 27]. SRS may also have a role as adjuvant therapy when subtotal resection is accomplished [26, 27]. SRS has been shown to be associated with a high tumor control rate with a low risk of adverse effects [27].

## 4. Conclusions

Optic nerve hemangioblastomas are unusual. However, hemangioblastoma should be considered in the differential diagnosis for optic nerve tumors when radiographic imaging shows evidence of increased vascularity. This allows the physician to perform appropriate pre-operative planning and counseling for the increased risks associated with surgical treatment of hemangioblastoma. Additionally, a high suspicion for VHL disease is necessary when a CNS hemangioblastoma is identified to address the causes of higher morbidity and mortality. Although not a common optic nerve tumor, hemangioblastoma should be considered in the differential diagnosis as the ramifications of surgical expectation and patient outcome could be drastically affected.
